# Contralateral Prophylactic Mastectomy in Women with Unilateral Breast Cancer Who Are Genetic Carriers, Have a Strong Family History or Are just Young at Presentation

**DOI:** 10.3390/cancers12010140

**Published:** 2020-01-06

**Authors:** Victoria Teoh, Marios-Konstantinos Tasoulis, Gerald Gui

**Affiliations:** Department of Breast Surgery, Royal Marsden NHS Foundation Trust, Fulham Road, London SW36JJ, UK; marios.tasoulis@rmh.nhs.uk (M.-K.T.); Gerald.Gui@rmh.nhs.uk (G.G.)

**Keywords:** contralateral prophylactic mastectomy, contralateral breast cancer, BRCA, CHEK2, PALB2, ATM, mutation carriers, family history, survival

## Abstract

The uptake of contralateral prophylactic mastectomy is rising with increasing trends that are possibly highest in the USA. Whilst its role is generally accepted in carriers of recognized high-risk predisposition genes such as *BRCA1* and *BRCA2* when the affected individual is premenopausal, controversy surrounds the benefit in less understood risk-profile clinical scenarios. This comprehensive review explores the current evidence underpinning the role of contralateral prophylactic mastectomy and its impact on contralateral breast cancer risk and survival in three distinct at-risk groups affected by unilateral breast cancer: known genetic carriers, those with strong familial risk but no demonstrable genetic mutation and women who are of young age at presentation. The review supports the role of contralateral prophylactic mastectomy in “high risk” groups where the evidence suggests a reduction in contralateral breast cancer risk. However, this benefit is less evident in women who are just young at presentation or those who have strong family history but no demonstrable genetic mutation. A multidisciplinary and personalized approach to support individuals in a shared-decision making process is recommended.

## 1. Introduction

The incidence of women with breast cancer who elect to undergo contralateral prophylactic mastectomy is steadily increasing, with preponderance amongst Caucasians, young women, and those with a higher socioeconomic status [1,2]. A study of 496,488 women with unilateral Stage I–III breast cancer, from the Surveillance, Epidemiology, End Results (SEER) Program database demonstrated an increase in contralateral prophylactic mastectomy rates performed for unilateral invasive breast cancer from 3.9% in 2002 to 12.7% in 2012 [3]. This effect was reproduced in a National Cancer Database review of 553,593 patients, showing an increase in contralateral prophylactic mastectomies from 4.1% in 2003 to 9.7% in 2010. This finding was most marked in young women, where those <45 years (*n* = 73,888) showed an increase from 9.3% in 2003 to 26.4% in 2010 [4].

Factors that contribute to this decision include patient age, disease stage, previous breast biopsies, genetic predisposition or family history of breast cancer, fear of recurrence, concern with cosmetic symmetry and physician recommendation [1,5,6,7,8].

Patients tend to overestimate their risk of developing a contralateral breast cancer [9,10] as well as the extent of risk reduction conferred by contralateral prophylactic mastectomy [9,11]. Interestingly, whilst 43.9% of women with breast cancer considered contralateral prophylactic mastectomy, only 38.1% were aware that it did not improve survival, highlighting the importance of patient education [12].

Improvements in modern multidisciplinary management have led to a reduction in the incidence of contralateral breast cancer from approximately 0.6% to 0.2–0.5%/year [13]. Consequently, the role of contralateral prophylactic mastectomy and the context in which it is supported is debatable.

This comprehensive review explores the current evidence underpinning the role of contralateral prophylactic mastectomy and its impact on contralateral breast cancer risk and survival in three high-risk groups affected by breast cancer: (i) genetic carriers, (ii) strong family history with no demonstrable mutation, and (iii) young women.

## 2. Methods

A comprehensive literature review was performed, assessing all studies published in the English literature from 1974 to March 2019 across Embase and Medline search engines. Search terms “contralateral prophylactic mastectomy”, “unilateral breast cancer”, “*BRCA*”, “*TP53*”, “*PALB2*”, “*CHEK2*”, “*ATM*”, “mutation carrier”, “family history”, “young women”, “non-genetic carriers”, “overall survival”, “disease-free survival”, “contralateral breast cancer” and “risk” were included. Relevant references from identified papers were also included.

## 3. *BRCA 1*/*2* Carriers with Breast Cancer

### 3.1. BRCA 1/2 Carriers and Contralateral Breast Cancer Risk

*BRCA* carriers with breast cancer carry a higher risk of contralateral breast cancer, 23.7% (95% CI 17.6–30.5), compared with non-carriers, 6.8% (95% CI 4.2–10), respectively, (RR 3.56, 95% CI 2.50–5.08; *p* < 0.001). This risk was higher in *BRCA1* compared to *BRCA2* carriers (RR 1.42, 95% CI 1.01–1.99; *p* = 0.04) [14]. In a Dutch multicentre study of 6294 invasive breast cancer patients ≤50 years, the risk of contralateral breast cancer for *BRCA1*/*2* carriers at a median follow-up of 12.5 years was shown to be 2–3 times higher compared to non-carriers (HR 3.31, 95% CI 2.41–4.55; *p* < 0.001 and 2.17, 95% CI 1.22–3.85; *p* = 0.01 respectively). The 10-year cumulative contralateral breast cancer risk following the initial breast cancer diagnosis was 21.1% for *BRCA1*, 10.8% for *BRCA2* and 5.1% for non-carriers [15]. These findings were confirmed in a recent multicentre study where the 10-year cumulative risk was 25.1% (95% CI 19.6–31.9) for *BRCA1*, 13.5% (95% CI 9.2–19.1) for *BRCA2* and 3.6% (95% CI 2.2–5.7) for non-carriers [16].

The age of first breast cancer diagnosis is a significant predictor of contralateral breast cancer risk in *BRCA* carriers [17,18,19]. Risk estimates vary in the literature, ranging from 23.7–30.7% in young women (<40 years) across *BRCA1*/*2* carriers combined (*BRCA1*: 24–32%; *BRCA2*: 17–29%) [19,20,21,22,23,24]. This risk is lower in the >40 years age group, ranging from 8.4–21% (*BRCA1*: 11–52%; *BRCA2*: 7–18%) [15,17,19,20,21,22,24,25]. Similar results were shown in another study demonstrating a 10-year cumulative contralateral breast cancer risk of 23.9% (*BRCA1*: 25.5%; *BRCA2*: 17.2%) in patients <41 years, compared to 12.6% in the 41–49 year group (*BRCA1* 15.6%; *BRCA2* 7.2%) [15].

In a retrospective study of 1042 *BRCA1*/*2* carriers with breast cancer, Graeser demonstrated that the 25-year cumulative contralateral breast cancer risk for *BRCA1* carriers with the first breast cancer diagnosis at age <40years, 40–50 years and >50 years, was 62.9% (95% CI 50.4–75.4), 43.7% (95% CI 24.9–62.5) and 19.6% (95% CI 5.3–33.9) respectively. In *BRCA2* carriers, the corresponding rates were 63% (95% CI 32.8–93.2), 48.8% (95% CI 22.7–74.9) and 16.7% (95% CI 1.0–32.4) for the respective age groups [19].

### 3.2. Contralateral Prophylactic Mastectomy and Risk of Contralateral Breast Cancer

Contralateral prophylactic mastectomy reduces the risk of contralateral breast cancer in *BRCA* mutation carriers [14,26,27]. This risk reduction has been reported to be in the range of 91% [27]. This is further supported by a meta-analysis showing that contralateral prophylactic mastectomy resulted in a 93% reduction in contralateral breast cancer risk (RR 0.072; 95%CI 0.035–0.588) [26].

### 3.3. Contralateral Prophylactic Mastectomy and Survival

There is conflicting evidence on whether contralateral prophylactic mastectomy improves survival in *BRCA* carriers with breast cancer [14,26,27,28,29,30,31] (Table 1). In a multicenter, retrospective study of 242 *BRCA* carriers with breast cancer, contralateral prophylactic mastectomy was associated with improved overall survival on multivariate analysis, having adjusted for risk-reducing salpingo-oophorectomy (HR 0.49, 95% CI 0.29–0.82) [30]. Similar findings have been reported in other cohort studies [27,29,30,31].

In a retrospective study by Van Sprundel, contralateral prophylactic mastectomy was associated with superior overall survival compared to active surveillance at 5-year follow up (94% vs 77%, *p* = 0.03). However, this difference was not significant once adjusted for prophylactic oophorectomy (HR 0.35, *p* = 0.14) [27]. Notably, Metcalfe observed a survival benefit only in the second decade of follow-up following initial breast cancer diagnosis (HR 0.52, 95% CI 0.29–0.93) but not during the first 10 years of follow-up (HR 0.65, 95% CI 0.34–1.22) [29].

A meta-analysis by Valachis demonstrated no difference in breast-cancer-specific survival between *BRCA* carriers who underwent contralateral prophylactic mastectomy against those who did not (HR 0.78, 95% CI 0.44–1.39; *p* = 0.40) [14]. However, a meta-analysis including two additional studies demonstrated a decrease in “all-cause” mortality [26]. To further add to the ambiguity, a recent prospective study showed that contralateral prophylactic mastectomy conferred no benefit in 5-year overall survival between *BRCA* carriers and non-carriers with triple negative breast cancer [32]. The available findings should be interpreted with caution as they are mostly based on retrospective studies that may contain recognized and unrecognized biases.

## 4. “Other” Genetic Carriers (*CHEK2*, *TP53*, *ATM*, *PALB2*, *PTEN*, *CDH1*) with Breast Cancer

Mutations in *CHEK2*, *TP53*, *ATM*, *PALB2*, *PTEN*, and *CDH1* account for a small fraction of familial breast cancers. The available studies are sparse and primarily family-based, with potential ascertainment bias. It should be noted that the existing literature focusses mainly on relative rather than absolute risk estimates.

### 4.1. “Other” Genetic Carriers and Contralateral Breast Cancer Risk

#### 4.1.1. CHEK2 Mutation Carriers and Contralateral Breast Cancer Risk

In a recent meta-analysis, Akdeniz demonstrated an increased contralateral breast cancer risk for CHEK2* 1100delC carriers (RR 2.75, 95% CI 1.77–4.27) [33]. This mutation is associated with bilateral disease and an increased risk of bilateral breast cancer which varies between two to six-fold [15,34,35,36,37,38,39,40] (Table 2). It has been suggested that *CHEK2* carriers may be more sensitive to ionizing radiation that may contribute to contralateral breast cancer rates in patients receiving adjuvant radiotherapy following breast conserving surgery [40,41]. The true impact of radiation in this context is questionable as consistently increased contralateral breast cancer risk has been demonstrated in patients treated with or without radiotherapy (HR 4.12, 95% CI 2.49–6.83 and HR 3.17, 95% CI 1.36–7.35, respectively) [39,41].

#### 4.1.2. TP53 Mutation Carriers and Contralateral Breast Cancer Risk

There are no studies estimating contralateral breast cancer risk in *TP53* carriers with breast cancer.

#### 4.1.3. ATM Mutation Carriers and Contralateral Breast Cancer Risk

In a multicentre, population-based, case-control study, Concannon suggested that four common variants of *ATM (c.1899-55T>G; c.3161C>G; c.6348-54T>C and c.5558A>T)* were associated with a lower contralateral breast cancer risk (overall RR 0.8, 95% CI 0.6–0.9) compared to those with rare, missense *ATM* mutations. The protective mechanisms may occur through an alteration in *ATM* activity as an initiator of DNA damage response or through its role in TP53 regulation [42]. Bernstein suggested that common *ATM* variants may exert a protective effect and reduce contralateral breast cancer risk, while rare *ATM* missense, deleterious variants may act synergistically with radiation exposure to increase this risk [43]. In this study, the variants: *c.1899-55T>G* (RR 0.5, 95% CI 0.3–0.8), c.3161C>G (RR 0.5, 95% CI 0.3–0.9), *c.5558A>T* (RR 0.2, 95% CI 0.1–0.6), and *c.6348-54T>C* (RR 0.2, 95% CI 0.1–0.8) were associated with significantly reduced risk. On the other hand, female carriers of any rare missense *ATM* variant, who received radiation therapy for their first breast cancer, had a significantly elevated contralateral breast cancer risk compared to unexposed women (RR = 2.8 for <1.0 Gy dose and RR = 3.3 for ≥1.0 Gy dose to the contralateral breast).

The direct relationship between the presence of *ATM* variants and the overall risk of contralateral breast cancer remains controversial, although the combination of radiotherapy and certain *ATM* missense variants appears to accelerate tumour development [44].

#### 4.1.4. PALB2 Mutation Carriers and Contralateral Breast Cancer Risk

There are no studies estimating contralateral breast cancer risk in *PALB2* carriers with breast cancer.

#### 4.1.5. CDH1 Mutation Carriers and Contralateral Breast Cancer Risk

There are no studies on the risk of contralateral breast cancer in *CDH1* carriers.

#### 4.1.6. PTEN Mutation Carriers and Contralateral Breast Cancer Risk

There are no studies estimating contralateral breast cancer risk in *PTEN* carriers with breast cancer.

### 4.2. Contralateral Prophylactic Mastectomy and Risk of Contralateral Breast Cancer

No studies have investigated the role of contralateral prophylactic mastectomy in the risk reduction of contralateral breast cancer in patients with a diagnosis of breast cancer that harbor a genetic mutation in non-*BRCA1*/*2* genes (*CHEK2*, *TP53*, *ATM*, *PALB2*, *CDH1* and *PTEN*).

### 4.3. Contralateral Prophylactic Mastectomy and Survival

There is no data to support any survival benefit from contralateral prophylactic mastectomy in this group of patients (“other” genetic carriers). This would be even more challenging in those with *TP53*, *CDH1 and PTEN* mutations because of the additional competing cancer risk. In view of the limited evidence, no further comment can be made, except to reinforce that contralateral prophylactic mastectomy should be considered on an individual basis for women with unilateral breast cancer in this group.

## 5. Familial Breast Cancers with no Demonstrable Genetic Mutations

### 5.1. Familial Breast Cancers with No Demonstrable Genetic Mutation and Contralateral Breast Cancer Risk

A positive family history remains a strong risk factor for contralateral breast cancer, even after excluding mutation carriers [46,47,48]. Table 3 summarizes the current literature on the impact of positive family history on contralateral breast cancer risk and survival. In a multicentre, population-based, case-control study of 1521 contralateral breast cancer cases against 2212 matched controls of unilateral breast cancer, Reiner demonstrated that non-mutation carriers with any 1st or 2nd degree relative of breast cancer had a nearly two-fold increased contralateral breast cancer risk (RR 1.8, 95% CI 1.3–2.4), compared to individuals without a family history. This risk is similar to that shown in previous studies [49,50]. In this non-mutation carrier group, a 1st degree family history of bilateral breast cancer increased the contralateral breast cancer risk by more than three-fold (RR 3.4, 95% CI 1.5–7.4). Where there is only an affected 2nd degree relative, the individual is at a 40% increased risk compared to an individual without a family history. The 10-year absolute contralateral breast cancer risk in non-mutation carriers with a 1st or 2nd degree family history is 8.3% (95% CI 5.5–12.6) and 6.6% (95% CI 4.4–10) respectively [46].

In a retrospective study of 6230 women from high risk families, with or without a known *BRCA1*/*2* mutation, Rhiem observed a cumulative contralateral breast cancer risk, 25 years after a first breast cancer diagnosis of 44.1% (95% CI 37.6–50.6) in *BRCA1* positive families, 33.5% (95% CI 22.4–44.7) in *BRCA2* positive families and 17.2% (95% CI 14.5–19.9) in *BRCA1*/*2* negative families [56]. This effect was previously demonstrated in smaller cohort studies linking a higher contralateral breast cancer risk with a family history with and without a young age of first breast cancer diagnosis [51,53].

The age at which the affected relative is diagnosed with their first breast cancer and the presence of bilateral disease impacts on contralateral breast cancer risk. Rhiem further observes that patients diagnosed with breast cancer at age <40 years had a cumulative risk 25 years from primary diagnosis of 55.1% and 38.4% for *BRCA1* and *BRCA2*-positive family history, respectively. The corresponding risk was 28.4% in patients from non-*BRCA* families [56].

The highest risk lies with women who have relatives with early-onset, bilateral breast cancer [52,53,54,57]. The 10-year absolute risk in individuals whose 1st degree relative received a unilateral breast cancer diagnosis at a young age (<40 years) is similar to that of an individual with a 1st degree relative diagnosed with bilateral breast cancer (13.5% and 14.1% respectively). When there was a combination of a family history of a 1st degree relative, an affected relative with bilateral breast cancer or at a young age (<40 years), the 10-year contralateral breast cancer risk increased significantly to 36% [46]. A similar cumulative risk of contralateral breast cancer by the age of 80 (32%, 95% CI 13–66) was observed in a study of 78,775 breast cancer patients, with a maternal history of bilateral breast cancer [53].

### 5.2. Contralateral Prophylactic Mastectomy and Risk of Contralateral Breast Cancer

Contralateral prophylactic mastectomy may reduce the risk of contralateral breast cancer in women with an elevated genetic or familial risk [21]. This meta-analysis demonstrated a risk reduction in women with *BRCA*-positive families (HR 0.03; *p* = 0.0005). However, only 4% (19/430) of the cohort were non-carriers, with the remaining 96% representing mutation carriers. Fayanju reported a significant reduction in pooled relative (RR 0.04, 95% CI 0.02–0.09) and absolute risk (−24%, 95% CI (−35)–(−12.4)) of metachronous contralateral breast cancer amongst recipients of contralateral prophylactic mastectomy [58]. This analysis included studies with a significant proportion of BRCA carriers which may lead to an overestimation of risk. A case-control study of women with stage I/II breast cancer and a positive family history reported a 95% decreased risk (HR 0.05, 95% CI 0.01–0.19; *p* < 0.0001) of contralateral breast cancer following contralateral prophylactic mastectomy, at a median follow-up of 17.3 years, compared to a matched cohort of women who did not receive mastectomy. However, this cohort, with either an affected 1st or 2nd degree relative was not screened for mutation status [55].

McDonnell also demonstrated a contralateral breast cancer risk reduction following contralateral prophylactic mastectomy in pre- and postmenopausal women with a strong family history of breast/ovarian cancer i.e., 94.4% (95% CI 87.7–97.9) and 96% (95% CI 85.6–99.5) respectively, at a median follow up of 10 years using the Anderson model [59] to predict the risk [60]. Although the cohort had a strong family history, the patients had not been screened for mutations. Similar to studies with undefined gene carriers within the study population, this data should be interpreted with caution as the effect from contralateral prophylactic mastectomy may be overestimated from competing risks conferred by mutation carriers.

### 5.3. Contralateral Prophylactic Mastectomy and Survival

The evidence on the effect of contralateral prophylactic mastectomy on disease-free and overall survival is conflicting (Table 4).

A Cochrane review of 1708 women with variable familial risk, who underwent contralateral prophylactic mastectomy, concluded that although this decreased the incidence of contralateral breast cancer, there was no association with survival improvement [61]. The meta-analysis conducted by Fayanju demonstrated no association with breast-cancer-specific and overall survival, despite a reduction in the risk of distant metastases or recurrence [58]. The lack of survival benefit from contralateral prophylactic mastectomy in breast cancer patients with elevated familial risk is also reported in smaller, retrospective cohort studies [27,62,63] but with notable exceptions. Boughey reported improved overall (HR 0.77, 95% CI 0.60–0.98; *p* = 0.03) and disease-free survival (HR 0.67, 95% CI 0.54–0.84) on multivariate analysis [55]. In a review of 908 patients receiving against 46,368 not receiving contralateral prophylactic mastectomy, Herrinton demonstrated that mastectomy reduced breast cancer mortality (HR 0.57, 95% CI 0.45–0.72) and overall mortality (HR 0.6, 95% CI 0.5–0.72) across all levels of familial risk [64]. Furthermore, Davies demonstrated that young women (<40 years) with unilateral, stage I disease and a 1st degree relative with bilateral breast cancer, were the only group to have a quality-adjusted life year benefit from contralateral prophylactic mastectomy, which was similar to that of a *BRCA1*/*2* carrier [63].

## 6. Young Women with Breast Cancer

### 6.1. Young Women with Breast Cancer and Contralateral Breast Cancer Risk

The definition of ‘young’ age group in the literature, varies from the “under-35”- to 50 years. Young age at first primary breast cancer diagnosis is associated with an increased contralateral breast cancer risk, poor prognosis and serves as an independent predictor of recurrence and breast-cancer-related death [66,67,68,69,70,71] (Table 5). Older studies did not account for *BRCA* mutation carriers, which may confound contralateral breast cancer risk and survival. Furthermore, they do not consider risk-reducing adjuvant therapies. In a retrospective study of 652 patients ≤35 years compared to 2608 women >35 years, the relative risk of contralateral breast cancer was 2.48 in the younger, compared to the older group [70]. This finding is supported by Li, who demonstrated an increased HR of 2.8 (95% CI 1.1–6.9), 2.1 (95% CI 1.1–4.4) and 1.9 (95% CI 1.1–3.5) in the ≤29 years, 30–34 years and 35–39 years age groups, compared to women diagnosed at age ≥40 [67]. The contralateral breast cancer risk is further elevated in HER2-overexpressing and triple negative subtypes [70].

### 6.2. Contralateral Prophylactic Mastectomy and Risk of Contralateral Breast Cancer

The younger age group is generally underrepresented in studies evaluating the role of contralateral prophylactic mastectomy. Using a Surveillance, Epidemiology, End Results database analysis of 107,106 women, of whom 8902 (8.3%) underwent contralateral prophylactic mastectomy, Bedrosian conducted a subgroup analysis of young women (<50 years) and the risk of contralateral breast cancer after contralateral prophylactic mastectomy, in both ER-negative and ER-positive, early-stage breast cancer. In ER-positive disease, the cumulative incidence of contralateral breast cancer during the 6-year study period was 0.13% vs. 0.46% (*p* = 0.07) in the contralateral prophylactic mastectomy vs. non-mastectomy group, and in ER-negative disease, 0.16% vs. 0.90% (*p* = 0.05) respectively [74]. These results should be interpreted with caution though as the study population was not screened for genetic carriers and also patients with a strong family history were not excluded.

### 6.3. Contralateral Prophylactic Mastectomy and Survival

There is conflicting data on the impact of contralateral prophylactic mastectomy on survival in this patient group. In a population-based study of 614 women <35 years, 81 (13.2%) of whom were elected for contralateral prophylactic mastectomy, Bouchard-Fortier demonstrated that recurrences, defined as local, regional or distant, were significantly fewer for patients with contralateral prophylactic mastectomy than without (32.1% vs. 52.9%, *p* < 0.001; HR 0.61; *p* = 0.02). However, this did not translate to an improvement in breast cancer-specific survival [75].

In an analysis of the National Cancer Database between 2004 and 2014, Lazow demonstrated that after controlling for patient demographics, tumor grade and use of adjuvant therapies, bilateral mastectomy in women < 40 years was associated with increased 10-year overall survival (HR 0.75, 95% CI 0.59–0.96; *p* = 0.023), compared to the unilateral mastectomy group [76]. This trend was also observed in a preceding National Cancer Database review from 1998–2002, demonstrating a 5-year overall survival benefit of 2% in young patients (adjusted HR 0.88, 95% CI 0.83–0.93; *p* < 0.001) between these two groups [78]. In a retrospective study of 42/481 (8.73%) young women <40 years, who were elected for contralateral prophylactic mastectomy, Zeicher reported that this was associated with improved 10-year overall survival (HR 2.35, 95% CI 1.02–5.41; *p* = 0.046), although this effect was not demonstrable for 5-year overall survival [71].

There is a suggestion that contralateral prophylactic mastectomy may confer benefit in young women with early-stage, ER-negative breast cancer. In a population-based study of 107,106 breast cancer patients, 3731 (3.48%) of whom were young (18–49 years), contralateral prophylactic mastectomy was associated with improved disease-specific mortality (HR 0.68, 95% CI 0.53–0.88; *p* = 0.004). This effect was not reproduced in young women with early-stage, ER-positive breast cancer [74].

Other retrospective cohort or population-based studies refute the survival benefit of contralateral prophylactic mastectomy in young women [72,73,75,77]. In a review of 9044 young women (<40 years) with breast cancer, Chen demonstrated no improvement in overall or breast cancer-specific survival [2]. This was supported in a retrospective study of 10,226 patients with invasive lobular carcinoma, demonstrating no overall survival benefit from contralateral prophylactic mastectomy in the 18–50 years group, at a median follow up of 6.9 years [72]. Moreover, in a review of 14,627 women and at median follow-up of 6.1 years, having matched for tumour size/grade, ER status and nodal status, Pesce demonstrated that contralateral prophylactic mastectomy offered no overall survival benefit, in women aged <45 years, with stage I/II breast cancer (HR 0.93, *p* = 0.39) [73].

Overall, these findings should be interpreted with caution as the quality of the data does not allow for definitive conclusions to be drawn.

## 7. Discussion

Contralateral prophylactic mastectomy is increasingly being performed despite an ambiguity of evidence to support an oncological benefit. In 2007 and 2009, two studies reported that contralateral prophylactic mastectomy rate had increased 148% and 150% among all patients diagnosed with non-invasive and invasive breast cancer respectively [79,80]. Current trends in the U.S.A show an absolute percentage increase in the range of 25% [81]. This trend is modest in European studies suggesting a difference in practice and healthcare environments [82,83,84]. Nonetheless, this increased utilization of contralateral prophylactic mastectomy is a cause of concern for clinicians because of the associated surgical risks, complications, and psychological and financial burden in the absence of robust evidence to support significant oncological benefits.

Although intuitively it is expected that contralateral prophylactic mastectomy would decrease the risk of contralateral breast cancer, the available data only support this in patients with *BRCA1*/*2* gene mutations [14,26,27]. In women with strong family history or young age at diagnosis, the effect of contralateral prophylactic mastectomy is less well studied and the existing literature should be interpreted with caution because of the potential biases. At present, there are no models that allow for calculation of contralateral breast cancer risk in a polyfactorial model. Such a model might be useful in stratifying risk and aiding physicians to provide precise and unbiased estimation of risk, in order to offer individualized counselling to patients, inform decision-making and mitigate patient overestimation of cancer risk which may drive unnecessary surgery.

Despite the potential decrease in contralateral breast cancer, the effect of contralateral prophylactic mastectomy on oncological outcomes is debatable as studies suggest that this reduction is not translated into survival benefit. Moreover, the role of contralateral prophylactic mastectomy per se as a contributing factor for improved outcomes in women with unilateral breast cancer is difficult to accurately define, as the majority of the available data is of limited quality. Meta-analyses are only as strong as the independent studies they comprise. The majority of studies are retrospective cohorts and based on population/family studies. Therefore, the results should be interpreted with caution because of potential uncontrolled biases. The way to address these issues is with higher quality data but it is unlikely that the future will harbour randomized clinical trials investigating the impact of contralateral prophylactic mastectomy on contralateral breast cancer risk and survival due to patient preference and ethical considerations. One proposal is to set up robust prospective registries to help enhance our knowledge in the field. The majority of existing studies do not account for the significant role conferred by improved systemic therapies and its effect on contralateral breast cancer risk and improved oncological outcomes, factors that merit future research.

Recently, and in order to aid clinicians approach this controversial topic, both the American Society of Breast Surgeons and Association of Breast Surgery published consensus statements on the utilization of contralateral prophylactic mastectomy. Both were aligned on supporting its use in women with significant contralateral breast cancer risk i.e., *BRCA1*/*2* mutations, patients with a history of mantle field radiation to the chest before age 30 years [85,86]. However, a multidisciplinary, individualised approach is required to help women in their informed decision-making process.

## 8. Conclusions

In conclusion, contralateral prophylactic mastectomy may be supported in ‘high-risk’ groups as evidence indicates a possible reduction in contralateral breast cancer risk and also potentially improved oncological outcomes. The evidence to demonstrate that this may confer benefit in the other risk groups or in older patients is less established. It is therefore imperative to follow a multidisciplinary, personalised approach, to educate women on the best available evidence and to support individuals in a shared-decision making process.

## Figures and Tables

**Table 1:** Studies looking at the impact of CPM on CBC risk and survival in *BRCA1*/*2* mutation carriers.

Author	Year	Study Type	Follow up	Patient	Findings
Li [26]	2016	Meta-analysis (2 RC/1 PC/1 RCC)	n/a	4/4574 studies (1672 individuals)	CPM significantly decreased CBC risk in *BRCA1*/*2* mutation carriers (RR 0.072; 95% CI, 0.035–0.148). CPM is associated with a decrease in “all–cause” mortality (HR 0.512; 95% CI 0.368–0.588)
Valachis [14]	2014	Meta-analysis (1 RCC/1 RC)	n/a	2/13 studies	CPM was not associated with a benefit in BCSS HR 0.78 (95% CI 0.44–1.39, *p* = 0.40)
Copson [32]	2018	Prospective cohort	Median 8.2 years	21 *BRCA* carriers/10 non-carriers, with TNBC	CPM conferred no difference in 5-year OS between *BRCA* carriers and non-carriers with TNBC 83% (95% CI 74–89) vs. 74% (95% CI 69–78) HR 0.98 (95% CI 0.58–1.65), *p* = 0.94
Heemskerk-Gerritsen [30]	2015	Multicentre retrospective cohort	Median 11.4 years	242/583(52%) carriers with BC who underwent CPM	CPM improved OS HR 0.49 (0.29–0.82)
Metcalfe [29]	2014	Retrospective observational	Median follow up 14.3 yrs (0.1–20.0)	390 *BRCA1/BRCA2* carriers with a positive family history	At 20 years follow up, CPM was associated with a 48% reduction in death from breast cancer (HR 0.52; *p* = 0.03). * Not significant on propensity score adjusted analysis
Evans [31]	2013	Retrospective case-control	Median 9.7 years	105/698 (15%) *BRCA 1/2* carriers with BC who underwent CPM	CPM improves OS 89% (CPM) vs. 71% (non-CPM) at 10 year follow up (*p* < 0.001)
Van Sprundel [27]	2005	Retrospective cohort	Mean 3.5 years	69/148 (47%) BRCA 1/2 carriers with BC who underwent CPM	CPM reduced the risk of CBC in BRCA1/2 carriers by 91% No significant difference in OS between CPM and non-CPM group HR 0.35, *p* = 0.14 (adjusted for prophylactic oophorectomy)
Brekelmans [28]	2007	Retrospective case-control	Median 4.3 years	260 BRCA 1/2 carriers with BC vs. 759 non-carriers	CPM conferred no difference in BCSS HR 0.98 (95% CI 0.5–0.91, *p* = 0.96)

RC: retrospective cohort; RCC: Retrospective case-control; PC: prospective cohort; BCCS: breast-cancer-specific; OS overall survival; CPM: contralateral prophylactic mastectomy; BC: breast cancer; CBC: contralateral breast cancer; TNBC: triple negative breast cancer; HR: hazard ratio.

**Table 2:** Studies looking at CBC risk and survival in CHEK2* 1100delC mutation carriers.

Author	Year	Study	Median Follow up	N	Findings
Akdeniz [33]	2019	Meta-analysis	N/A	68 studies	CBC risk by mutation carriers *BRCA1* RR 3.7 (95% CI, 2.8–4.9) *BRCA2* RR 2.8 (95% CI, 1.8–4.3) CHEK2* 1100delC RR 2.7 (95% CI, 2.0–3.7)
Kriege [39]	2014	Retrospective, multicentre cohort study	6.8 years	193/4722 (4.1%) BC patients with CHEK2* 1100delC mutation	Higher risk of CBC HR 3.97 (95% CI 2.59–6.07) 10-year risk of CBC is 28.9%
Weischer [37]	2012	Meta-analysis	6 years	459/25571 (1.8%) BC patients with CHEK2* 1100delC mutation	20-year cumulative risk of developing BC is 25–30% (HR 3.52) No comment on CBC rates
Mellemkjaer [40]	2008	Population based, multicentre cohort study	N/A	17/2103 (0.8%) BC patients with CHEK2* 1100delC	No significant association between CHEK2* 1100delC mutation and CBC
Broeks [41]	2004	Case study	N/A	15/233 (6.4%) CHEK2* 1100delC mutation carriers with BBC 2/191 (1%) CHEK2* 1100delC mutation carriers with UBC	Increased risk of CBC in carriers OR 6.5 (95% CI 1.5–28.8, *p* = 0.005)
Schmidt [36]	2007	Retrospective cohort study	Median 10.1 years	54/1479 (3.7%) pre-menopausal BC patients with CHEK2* 1100delC mutation	CHEK2* 1100delC mutation carriers: Increased risk of ipsilateral second breast cancer HR 2.1 (95% CI 1.0–4.3; *p* = 0.049) Increased risk of CBC HR 1.7 (95% CI 1.2–2.4) Worse breast-cancer-specific survival HR 1.4 (95% CI 1.0–2.1; *p* = 0.072) Worse recurrence- free survival HR 1.7 (95% CI 1.2–2.4; *p* = 0.06)
De Bock [45]	2004	Prospective cohort	Median 3.8 years	34 BC patients with CHEK2* 1100delC mutation; 102 BC patients with no mutation	Compared to non-carriers, CHEK2* 1100delC mutation carriers: Increased risk of CBC compared RR = 5.74 (95% CI 1.67–19.65) Increased risk of distant metastasis RR 2.81 (95% CI 1.2–6.58) Worse DFS RR = 3.86 (1.91–7.78) No difference in overall survival. Mutation carriers more frequently had a 1st or 2nd degree female relative with breast cancer (*p* = 0.03)

BC: breast cancer; CBC: contralateral breast cancer; BBC: bilateral breast cancer; UBC: unilateral breast cancer; RR: relative risk; DFS: disease-free survival; ER: oestrogen receptor; HR: hazard ratio.

**Table 3:** Studies looking at the impact of positive FH of breast cancer on CBC rates, disease-free and overall survival.

The Impact of Positive FH of Breast Cancer on CBC Rates, Disease-Free and Overall Survival					
Author	Year	Study Type	Follow up	Patients	Findings
Reiner [46]	2018	Multicentre, population-based, case-control study	Not stated	1521 CBC cases with 2212 UBC controls	A 1st degree relative with BC confers increased risk of CBC RR 1.9 (95% CI 1.6–2.3) A 1st degree relative with BBC confers the highest risk of CBC RR 3.4 (95% CI 2.4–5) A 2nd degree relative increases the risk of CBC RR 1.4 (95% CI 1.2–1.7) Any 1st degree relative with breast cancer confers a 10-year AR of developing CBC of 8.1% (95% CI 6.7–9.8). The 10-year AR increases to 13.5% (95% CI 8.8–20.8) if this relative was <40 years at age of diagnosis. The 10-year AR is highest at 36% (95% CI 14.5–90.5) if the first degree relative was diagnosed with BBC at age <40 years. On subgroup analysis and exclusion of mutation carriers i.e., *BRCA*, *ATM*, *PALB2* and *CHEK2*, the increased 10-year AR associated with a 1st degree relative and a 1st degree relative with BBC remained significant similar to above-reported.
Kuchenbaecker [20]	2017	Prospective, multicentre, cohort study	Median 4 years (2–7)	3886 eligible for breast cancer analysis *BRCA1* (*n* = 2276); *BRCA2* (*n* = 1610)	Increased risk if ≥two 1st or 2nd degree relatives with breast cancer compared to no family history of BC; HR 1.99 Did not evaluate the effect of FH on CBC risk
Bernstein [51]	1992	Prospective cohort study	Mean 52 months	136/4550 (2.9%) patients with CBC and varying familial risk	Compared with no FH of breast cancer: Increased risk of CBC ~2x with a 1st degree relative with BC Increased risk of CBC ~3x if 1st degree relative was diagnosed at a young age (<35 years)
Ji [52]	2007	Population based, national database study	Not stated	56190 invasive and 6841 in situ BC patients	The risk of metachronous CBC measured by SIRs was higher with primary in situ disease compared to invasive cancer. SIR for metachronous CBC in women diagnosed with invasive BC: <45 years: 5.12 (95% CI 4.47–5.85) 45–55 years: 1.95 (95% CI 1.76–2.16) >55 years: 1.49 (95% CI 1.37–1.61) SIR for metachronous CBC in women diagnosed with 1st invasive BC and have: A positive FH 2.74 (95% CI 2.3–3.23) No FH 1.85 (95% CI 1.75–1.96) SIR for metachronous CBC in women diagnosed with in situ disease: <45 years: 5.12 (95% CI 4.47–5.85) 45–55 years: 1.95 (95% CI 1.76–2.16) >55 years: 1.49 (95% CI 1.37–1.61)
Narod [53]	2016	Population based, national database study	Not stated	4839 CBC patients out of 84819 patients with BC * (5.7%)	Young age at 1st BC diagnosis and a maternal cancer history increases the risk of CBC The 15-year cumulative risk of CBC was: 8.8% (95% CI 8.5–9.1) in the general population (regardless of maternal BC status) 12% (95% CI 11–13) in maternal UBC 13% (95% CI 9.5–17) in maternal BBC A maternal cancer history of UBC at an early age conferred the daughter a lifetime CBC risk of 35% (95% CI 25–46) * Mutation carriers not excluded as information not available from cancer registry
Vaittinen [54]	2000	Population based, national database study	Not stated	2529/72,092 (3.5%) CBC patients. 147 (5.8%) of CBC cases with 1st degree relative	Modest elevation in CBC risk for women with an affected 1st degree relative RR of 1.53
Boughey [55]	2010	Retrospective cohort	Median 17.3 years	385 patients with a positive FH; 385 matched controls	Patients with stage I or II BC and a positive family history who underwent CPM had: A 95% reduction in CBC rates; adjusted HR 0.05 (95% CI 0.01–0.19, *p* < 0.0001)

CPM: contralateral prophylactic mastectomy; BC: breast cancer; BBC: bilateral breast cancer; CBC: contralateral breast cancer; UBC: unilateral breast cancer; AR: absolute risk; SIR: Standardized incidence ratio; FH: family history; RD: risk difference; HR: hazard ratio.

**Table 4:** Studies looking at the impact of CPM on CBC and survival in BC patients with elevated familial risk.

Author	Year	Study Type	Follow up	Patients	Findings
Akdeniz [33]	2019	Meta-analysis	N/A	68 studies	A positive FH of BC was associated with increased CBC risk RR = 1.72 (95% CI 1.15–2.57)
Engel [16]	2019	Multicentre, prospective cohort study	Median 2.9 years	667 *BRCA1* carriers, 402 *BRCA2* carriers 1924 *BRCA1*/*2* noncarriers (*BRCA1*/*2*-negative families)	10-year cumulative CBC risk for *BRCA1*/*2* non carriers 3.6% (95 CI 2.2–5.7) Women with ≥2 relatives with BC had an increased risk of CBC, compared to women without any relative affected by BC HR 2.35 (95% CI 1.21–4.55) ER-negativity was not associated with an increased CBC risk in *BRCA1*/*2* non-carriers
Fayanju [58]	2014	Meta-analysis	N/A	14/79 studies	Patients with an elevated familial/genetic risk who had CPM (vs no CPM): Reduction in pooled RR of mCBC; RR 0.04 (95% CI 0.02–0.09; *p* < 0.001)) Reduction in pooled AR of mCBC; RD of −24% (95% CI −35.6 to −12.4; *p* = 0.013) Significant reduction in rates of distant/metastatic recurrence. CPM was not associated with improved OS or BCSS
Boughey [55]	2010	Retrospective cohort	Median 17.3 years	385 patients with a positive FH; 385 matched controls (parent, sibling or 2nd degree relative with BC) * no genetic screening	Patients with stage I/II BC and a positive family history who underwent CPM had: A 95% reduction in CBC rates; adjusted HR 0.05 (95% CI 0.01–0.19, *p* < 0.0001) Improved OS (HR 0.77 (95% CI 0.60–0.98, *p* = 0.03)) Improved DFS (HR 0.67 (95% CI 0.54–0.84))
McDonnell [60]	2001	Retrospective cohort	Median 10 years	745 BC patients (388 premenopausal (<50 yrs); 357 postmenopausal with a positive FH	CPM conferred a CBC risk-reduction: In premenopausal women of 94.4% (95% CI 87.7–97.9) In postmenopausal women of 96% (95% CI 85.6–99.5)
Herrinton [64]	2005	Retrospective cohort	Median 5.7 years	1072/50,000 BC patients undergoing CPM	Across all levels of familial risk, CPM: Reduces breast cancer mortality (HR = 0.57; 95% CI 0.45–0.72) Reduces overall mortality (HR = 0.6; 95% CI 0.5–0.72)
Peralta [65]	2001	Retrospective cohort	Mean 6.8 years	23/64 (36%) BC patients undergoing CPM and with ≥one affected 1st degree relative (not screened for mutations)	None of the patients undergoing CPM developed a subsequent CBC
Kiely [62]	2010	Retrospective cohort	Median 8 years	154/1018 women who underwent CPM, with FH of BC ± BRCA mutations	Reduced rate of CBC in women who underwent CPM with no apparent benefit in survival

CPM: contralateral prophylactic mastectomy; BC: breast cancer; CBC: contralateral breast cancer; mCBC: metachronous contralateral breast cancer; BBC: bilateral breast cancer; UBC: unilateral breast cancer; FH: Family history; RR: relative risk; AR: absolute risk; DFS: disease-free survival; OS: overall survival; BCSS: breast cancer-specific survival’ ER: oestrogen receptor; HR: hazard ratio.

**Table 5:** Studies looking at the impact of CPM on CBC and survival in young women with breast cancer.

Author	Year	Study Type	Median Follow up	Patient Demographics (Age, CPM Status)	Findings
Chen [2]	2019	Retrospective cohort	113 months	<35 years and CPM 811/3083 (26.3%) 35–39 years and CPM 1243/5961 (20.9%)	No difference in BCSS from CPM HR 1.209 (95% CI 0.908–1.610, *p* = 0.194) No difference in OS from CPM HR 1.179 (95% CI 0.902–1.540, *p* = 0.228)
Yu [72]	2018	Retrospective cohort	6.9 years	910/1806 young patients (18–50 years) with CPM	No difference in OS in women with a young age (18–50 years) who had CPM HR 0.93 (95% CI 0.70–1.24; *p* = 0.627)
Pesce [73]	2014	Retrospective cohort	6.1 years	4338/10,289 (29.7%) young women (<45 years) with Stage I/II cancer with CPM	CPM provides no survival benefit in young women (<45 years) Compared to unilateral mastectomy HR 0.93; *p* = 0.39 With early-stage (T1N0) breast cancer HR 0.85; *p* = 0.37 With ER-negative breast cancer HR 1.12; *p* = 0.32
Bedrosian [74]	2010	Population based cohort study	47 months	3731/27,336 (13.6%) young women (18–49 years) with CPM	CPM offers benefit in BCSS for young women (18–49 years) with early stage, ER-negative breast cancer HR 0.68 (95% CI 0.53–0.69), *p* < 0.001
Bouchard-Fortier [75]	2018	Population-based cohort	11 years	81/614 (13.2%) young women (≤35 years) with CPM	Risk of recurrence (breast/distant) was lower in the CPM group HR 0.61, *p* = 0.02 No difference in breast cancer-specific mortality from CPM HR 0.73 (95% CI 0.47–1.21)
Zeichner [71]	2014	Retrospective cohort	68 months	42/481 (8.73%) young women (<40 years) with CPM	CPM provides a benefit in 10-year overall survival * HR 2.35 (95% CI 1.02–5.41, *p* = 0.046) * effect not seen at 5-year overall survival
Lazow [76]	2018	Population-based cohort	Mean 62 months	4139/11,859 (34.9%) young women (<40 years) with CPM	CPM improves 10-year overall survival HR 0.75 (95% CI, 0.59–0.96) *p* = 0.023]
Park [77]	2017	Population based, national database study	Not stated	3648 DCIS patients <40 years (25.8% UM; 15.8% CPM)	No overall survival benefit from CPM compared to UM in the <40 years group

OS: overall survival; CPM: contralateral prophylactic mastectomy; unilateral mastectomy: BCSS: breast-cancer-specific survival.
